# Reply to Mangindaan, D. Comment on “Altaf et al. Non-Thermal Plasma Reduction of Ag^+^ Ions into Silver Nanoparticles in Open Atmosphere under Statistically Optimized Conditions for Biological and Photocatalytic Applications. *Materials* 2022, *15*, 3826”

**DOI:** 10.3390/ma17081751

**Published:** 2024-04-11

**Authors:** Noor Ul Huda Altaf, Shazia Shukrullah

**Affiliations:** Department of Physics, University of Agriculture Faisalabad, Faisalabad 38040, Pakistan

The authors of the mentioned paper are highly thankful to Dr. Dave Mangindaan for reproducing the statistical work of our paper and identifying a possible error in the regression Equation (3) in the published paper [1]. He proposed the following new regression Equation (3*), which may yield statistical data on the particle size in the last column of Table 1 that is closer to the empirical size.

(3*) $$\begin{array}{ll} Y\left(AgNPssize\right) & =31.53-2.16A-0.57B-3.78C-3.04A^{2}-3.39B^{2}-1.80C^{2}\\ & \;-1.56AB-0.73AC-0.38BC \end{array}$$

Considering the regression Equation (3*), proposed by Dr. Mangindaan, we rechecked our model and repeated the calculations. We concluded that the proposed regression equation may yield more acceptable results than the one reported in our work. However, we understand that the last two terms (−0.73AC−0.38BC) of the regression equation proposed by Dr. Mangindaan need correction. These terms should be positively signed (+0.73AC+0.38BC) for correctness of the data reported by Dr. Mangindaan in Table 1. By incorporating this correction, we present the following new regression Equation (3**). This Equation (3**) produces the particle sizes that best fit the data reported in Table 1.

(3**) $$\begin{array}{ll} Y\left(AgNPssize\right) & =31.53-2.16A-0.57B-3.78C-3.04A^{2}-3.39B^{2}\\ & -1.80C^{2}-1.56AB+0.73AC+0.38BC \end{array}$$

We also communicated with Dr. Mangindaan for comments and confirmation on Equation (3**) before finalizing our response to the journal. Dr. Mangindaan agreed that Equation (3**) produces the same values as predicted in the last column of Table 1 in our published article. We are highly thankful to Dr. Mangindaan for satisfactorily accepting our response to his feedback/comment on our published article.

When we were conducting this research work, we repeated the model against various independent variables to obtain a suitable regression equation. The equation given in the published paper was picked from several calculated equations. The finalized equation was somewhat similar to the one suggested by Dr. Mangindaan, with a change in the signs of the last two terms (please see Equation (3**)).

Dr. Mangindaan also suggested some typographical corrections in the published article [1] to support his comment, mostly mentioning that the scientific names in the list of references should be written in italics. These recommendations are appropriately identified as writing style and formatting adjustments rather than typographical errors. We used EndNote X7 for citing and referencing the literature according to the provided journal format and style. It is worth noting that such differences in referencing and styling are possible in scientific writing. Please note that while getting through the “ready to publish comment” of Dr. Mangindaan, we also noticed some typographical errors in it. These errors have been duly communicated to the Managing Editor for rectification prior to the online publication of the comment.

Dr. Mangindaan revised his original comment after having it peer-reviewed by the reviewers. Referring to one of the reviewers in his revised comment, he added some additional queries in the last paragraph on Page 3, regarding particle sizes and UV absorption peaks. As he mentioned in his comment, the respected reviewer was unsure about his opinion on UV absorption peaks since he was referring to a specific part of his work on commercially available AgNPs. The reviewer mentioned some UV absorption peaks from his work on AgNPs (20–80 nm), which he purchased from a third party. He did not mention the shape and purity of the nanoparticles, synthesis method, synthesis conditions, cleaning method, pH controlling method, stabilizer type, post-synthesis heat treatment, etc. [2,3,4,5,6]. All these conditions and parameters affect the UV absorption range, even in the same particle size range. We used a plasma–liquid interaction method to produce AgNPs under specific conditions. We used glucose as a stabilizing agent, which may have caused a capping effect and a shift in UV absorption. However, we agree with the query of the respected reviewer on the difference in particle sizes calculated through two different techniques in Table 4 of the published paper [1]. Previously, we processed SEM images using a MATLAB R2015a code, which may not have produced true particle size distribution, as shown in Figure 8 and Table 4 of the published paper [1]. Considering the feedback of the respected reviewer, we recalculated the particle size using specialized software. The SEM-based recalculated particle sizes are much smaller than those calculated using the coding method. XRD analysis gave the particle sizes of 19.89 nm and 33.45 nm for Run 4 and Run 6, respectively. The revised SEM analysis gives the particle sizes of 34.7 nm and 46.5 nm, respectively. There is still a notable difference between particle sizes calculated through these analyses since both techniques are based on completely different measurement methods. The published literature also mentioned such differences in sizes with different measurement techniques [6,7,8,9,10,11].

In conclusion, we are highly thankful to Dr. Mangindaan for pointing out this correction in the work. We endorse the regression Equation (3*) suggested by Dr. Mangindaan, with some changes in the last two terms. We have modified the regression equation (as Equation (3**)) by considering his suggestion and updated the predicted values in the last column of Table 1. Dr. Mangindaan also suggested some typographical corrections, mentioning that the scientific names in the list of references should be written in italics. These should not be addressed as typographical errors but as writing style and formatting corrections. While getting through the “ready to publish comment” of Dr. Mangindaan [12], we also noticed some typographical errors, which need to be corrected before publishing his comment. Dr. Mangindaan revised his original comment after having it peer-reviewed by the reviewers and added some additional queries on particle size and the UV absorption peaks of AgNPs. As mentioned in his comment, the respected reviewer was unsure about his opinion on UV absorption peaks since he was referring to a specific part of his work on commercially available AgNPs. However, we acknowledge the query of the respected reviewer on the difference in particle sizes calculated through two different techniques. The SEM-based recalculated particle sizes are much smaller than those reported in the paper. A difference in particle sizes, calculated through XRD and SEM analyses, is possible since both techniques follow completely different measurement mechanisms [7,8,9,10,11,13].

Finally, we thank the editorial team, Dr. Dave Mangindaan, and the reviewers for their time and effort.

## Figures and Tables

**Table 1:** Coded values with experimental and predicted responses obtained from a Box–Behnken design.

Runs	A	B	C	Experimental Size (nm)	Y (Size of NPs) (nm) Predicted Values
1	0	1	1	23.05	22.37
2	1	0	1	21.40	21.48
3	0	0	0	36.45	31.53
4	0	−1	1	19.89	22.74
5	−1	0	1	26.60	24.35
6	−1	0	−1	33.45	33.37
7	−1	1	0	25.33	28.26
8	1	−1	0	28.00	25.07
9	0	0	0	32.6	31.53
10	−1	−1	0	26.88	26.28
11	0	−1	−1	30.40	31.08
12	1	1	0	20.22	20.82
13	1	0	−1	25.33	27.58
14	0	0	0	25.55	31.53
15	0	1	−1	32.02	29.17
